# Cleft team strategy and caries experience in patients with a cleft lip and/or palate, a multicentre study

**DOI:** 10.1007/s40368-026-01226-7

**Published:** 2026-06-02

**Authors:** L. S. van der Knaap-Kind, Gisselle Carbajal Rodriguez, M. A. Schorer-Jensma, E. B. Wolvius, L. Kragt, N. C. W. van der Kaaij

**Affiliations:** 1 Department of Oral and Maxillofacial Surgery, Special Dental Care and Orthodontics, Erasmus University Medical Center, Rotterdam, Netherlands; 2 Stichting Bijzondere Tandheelkunde (SBT), Amsterdam, Netherlands; 3 Department of Orthodontics, Academic Center for Dentistry Amsterdam, Amsterdam, Netherlands; 4 Department of Oral and Maxillofacial Surgery, Amsterdam University Medical Centers, Amsterdam, Netherlands

**Keywords:** Dental caries, Paediatric dentistry, Restorative treatment, Cleft lip and/or palate

## Abstract

**Background:**

Most cleft teams in The Netherlands have a paediatric dentist as a permanent team member, but differences persist in patients receiving general or specialised dental care in-between team visits. The current study aims to find out if different strategies regarding dental care result in different caries experience and restorative care levels of cleft lip and/or palate (CL/P) patients.

**Methods:**

The cleft team of the Amsterdam University Medical Center, presented as Team Spec, refers all CL/P patients at age one to specialised dental care. The majority of CL/P patients treated in the Erasmus Medical Center, Rotterdam, receive dental care from a general dentist, Team Gen. Data on decayed teeth (dt), missing teeth (mt), filled teeth (ft), sum dmft and restorative care level for the primary dentition of CL/P patients were compared with independent t-tests. Negative binomial hurdle regression and generalised linear models were used to correct for age, gender and cleft type. No comparison of the costs of both strategies was made.

**Results:**

The restorative care level of Team Spec (51.50%) was significantly better than Team Gen (29.31%) (*p* < 0.001). Though sum dmft of both teams was comparable (*p* = 0.32), patients in Team Spec had fewer missing teeth due to caries (*p* = 0.01).

**Conclusion:**

Receiving specialised dental care is beneficial for the restorative care level of CL/P patients.

## Introduction

Though dental caries is one of the most widespread diseases in the world (Peterson 2005), children with a cleft lip and/or palate (CL/P) are even more vulnerable to developing caries compared to their non-CL/P peers (Antonarakis et al. 2013; Worth et al. 2017). Untreated and severely decayed teeth have a negative impact on children’s general health and wellbeing by causing discomfort, pain, sleeping problems, eating difficulties, learning disorders and odontogenic infections in the short term (Duijster et al. 2013; Grund et al. 2015; Leal et al. 2012; Moura-Leite et al. 2011; Souza et al. 2018). Further, early caries is a predictor for a low oral health-related quality of life in the long term (Kragt et al. 2016). The increased caries risk in CL/P patients can be explained by various associated congenital dental anomalies, such as microdontia, dilacerations, agenesis and supernumerary teeth, but also by the deviant anatomy and scarring of the lip and/or jaw, resulting in difficulty to maintain a good oral hygiene (Sa et al. 2016). Moreover, research has shown that CL/P patients more often experience dental fear and have limited coping behaviours in the dental setting (Vogels et al. 2011). This may complicate an effective curative treatment of an existing carious lesion.

The multidisciplinary cleft teams in the Netherlands are a good example of effective collaboration between medical and dental care professionals in the treatment of CL/P patients. The XXX cleft care guideline recommends that preferably a paediatric dentist should be a member of the cleft team (Cleft Guideline 2018). Subsequently, the Dutch cleft care guideline provides no recommendations regarding additional dental care visits. Since cleft team visits take place only once every 2–5 years, it is crucial that also the dental care outside the cleft team visit is well organized. Some cleft teams choose to refer all their patients to specialised paediatric dental care. However, strategies differ. Other cleft teams advise their patients to visit a general dentist. These patients are only referred for specialised dental care when untreated and/or inadequately treated carious lesions are observed during the cleft team visits and/or if parents are not satisfied with the received dental care.

Currently, it is unknown how the different strategies concerning dental care outside the cleft team impact the caries experience of the CL/P patients. Therefore, the aim of the present study is to compare dental caries status and restorative care levels of CL/P patients from two different cleft teams in the Netherlands, each employing a different strategy regarding dental care outside the cleft team. This comparison seeks to identify which approach is more beneficial for the caries experience of CL/P patients.

## Material and methods

This retrospective study was approved by the METC of the Amsterdam University Medical Center, Amsterdam, the Netherlands (2024.1036) and by the METC of the Erasmus Medical Center, Rotterdam, the Netherlands (MEC-2019-0535-A-0001).

### Study group

The study group was recruited from the cleft teams of the Amsterdam University Medical Center (AUMC), Amsterdam, the Netherlands, and the Erasmus Medical Center (Erasmus MC), Rotterdam, the Netherlands. The cleft teams of AUMC and Erasmus MC both have a paediatric dentist as a permanent cleft team member. As part of the cleft treatment, both teams record information on tooth brushing behaviours, frequency of dental visits and the experience of dental visits. Furthermore, an intra-oral inspection is a standard assessment during each cleft team visit in both teams. The main difference between the dental care approaches of the two cleft teams is the referral to either a general, or specialised dentist. At AUMC, the vast majority of the CL/P patients are standard referred to a specialised dentist during their first cleft team visit at age 1 year, with only a minority of CL/P patients declining this referral and instead visiting the general dentist. On the contrary, at Erasmus MC, the vast majority of the CL/P patients visit a general dentist. Only when untreated and/or inadequately treated carious lesions are observed during the cleft team visits and/or if the parents are not satisfied with the current dental treatments, a referral to a specialised dentist is made. In the results section the cleft team of AUMC will be described as ‘Team Spec’ and the team of Erasmus MCas ‘Team Gen’.

### Outcome variables

Dental caries is assessed with the Decayed Missing Filled Teeth (DMFT) index, a clinician-reported tool used commonly in epidemiological studies for the quantitative evaluation of the caries experience. During cleft team visits at AUMC and Erasmus MC, all CL/P patients, regardless of comorbidities, underwent an intra-oral examination in a dental chair. All patients with cleft lip and alveolus (CLA), cleft lip and palate (CLAP), or cleft palate (CP) were eligible for inclusion irrespective of comorbidities, as long as a reliable intraoral examination was feasible. Per patient, three components were recorded, generally by the paediatric dentist of the team: the number of teeth with carious lesions (decayed teeth, dt), the number of teeth missing due to caries (missing teeth, mt), and the number of teeth with restorations due to caries (filled teeth, ft). Collection of dmft data around the age of 5 years (range: 4–6 years) was standard practice at both AUMC and Erasmus MC, in alignment with the International Consortium for Health Outcomes Measurement (ICHOM) data set for cleft care. Following the protocol of each specific cleft team, Erasmus MC typically collects dmft scores around the age of 4 years, whereas at AUMC this information is collected during the cleft team visit around the age of 6 years. At AUMC there is no standard cleft team visit planned at age 4 years.

The dmft index distinguishes between the primary and permanent dentitions using lowercase letters (dmft, range 0–20) for the primary dentition. Dental caries status was calculated as the sum of decayed, missing, and filled teeth (dt + mt + ft).

Not only the caries experience, but also the restorative care level of each patient was quantified. This metric reflects the proportion of carious teeth that have received restorative treatment and is calculated using the formula: (ft/(dt + ft)) × 100%. The restorative care level was only calculated for patients with dmft > 0.

Data collection for the AUMC cleft team took place between April 2019 and December 2024. For the Erasmus MC cleft team, data were included from January 2016 to March 2025. In addition to caries-related outcomes, for the present study the following information was extracted from the patient records: age, gender and type of cleft.

### Data analysis

To test the reliability of dmft scoring during the cleft team, the intra-oral photographs of 20 randomly selected CL/P patients in AUMC were scored by a paediatric dentist (M.S.J.), resulting in good reliability of *κ* = 0.733. The re-scoring of the dmft by the paediatric dentist (L.S.K.K.) at Erasmus MC resulted in very good reliability of *κ* = 0.841. Both paediatric dentists, from AUMC and Erasmus MC, have much experience and are well equipped to assess dmft measurements.

Descriptive statistics were used to characterise the study population. Differences in gender and cleft type between the two cleft teams were analysed using Chi-square tests, whilst age at the moment of caries assessment was compared using an independent samples t-test. The dt, mt, ft, and dmft scores were reported as means with standard deviations, as well as absolute frequencies and percentages across three categories: 0, 1–3, and > 3. Mean differences in dt, mt, ft, and dmft scores between the two centres were assessed using independent t-tests, whilst differences in the categorical scores were analysed using Chi-square tests.

Due to overdispersion and zero-inflation of dmft, negative binomial hurdle regression (NBHR) was used to analyse the effect of treatment centre on caries prevalence (odds ratios, OR) and caries experience, in terms of number of teeth affected (rate ratios, RR). Two models were presented, the first adjusting for age and the second adjusting for age, gender and cleft type.

Associations between restorative care level and potential predictors were examined using a generalized linear model with a binomial distribution and logit link. In this model, the number of restored teeth was treated as the number of events, and the total number of affected teeth as the number of trials. The model included treatment centre, age, gender, and cleft type as predictors. Results were reported as OR with 95% confidence intervals.

Finally, the mean dmft scores of the two cleft centres were compared to reference data from the general population using one-sample *t*-tests. Van Meijeren-van Lunteren reported in 2021 a mean dmft = 0.97, SD = 2.17 in more than 4000 non-affected patients aged 6 years from the region of Rotterdam, the Netherlands, assessed between 2009–2012.

All statistical analyses were conducted using SPSS (version 28, IBM Corp., NY, USA) and R statistical software (R Foundation for Statistical Computing, Vienna, Austria). A *p* value of < 0.05 was considered statistically significant.

## Results

The description of the study population (*N* = 575) is presented in Table 1 and stratified for Team Spec (*N* = 212) and Team Gen (*N* = 363); in total, 338 male patients (58.8%) and 237 female patients (41.2%) were included in the study. Most of the patients had a CP (46.4%), followed by CLAP (37.9%) and CLA (15.7%). There was no difference in the prevalence of the cleft types between the two cleft teams (*χ*^2^(2, *N* = 575) = 0.28, *p* = 0.87), nor in gender (*χ*^2^(1, *N* = 575) = 0.97, *p* = 0.43). However, there was a significant difference in mean age at the moment of caries assessment of CL/P patients in Team Spec (*M* = 6.19, SD = 0.33) and in Team Gen (*M* = 4.52, SD = 0.88); *t*(573) = 26.69, *p* < 0.001.

**Table 1:** Basic characteristics of two cleft teams with different strategies concerning specialized dental care between team visits

	Team Spec (*n* = 212)	Team Gen (*n* = 363)	*p*-value
Mean age (SD)	6.19 (.33)	4.52 (.88)	< 0.001*
Gender			
Male	119 (56%)	219 (60%)	0.43
Female	93 (44%)	144 (40%)	
Cleft type			
CLA	31 (15%)	59 (16%)	0.87
CLAP	82 (39%)	136 (37%)	
CP	99 (47%)	168 (46%)	

*CP* cleft palate, *CLA* cleft lip and alveolus, *CLAP* cleft lip alveolus and palate

*p* < 0.05 is considered significant, *Significant difference based on Chi-square tests for gender and cleft type and independent *t*-test for age

There was no significant difference in mean dmft between the study groups (*M*_team spec_ = 2.03, SD = 3.27; *M*_team gen_ = 1.82, SD = 3.57; *t*(573) = 0.68, *p* = 0.84). However, there were significant differences in mean decayed teeth outcomes between Team Spec (*M* = 0.83, SD = 1.78) and Team Gen (M = 1.09, SD = 2.71); *t*(573) = − 1.26, *p* = 0.01; mean missing teeth outcomes between Team Spec (*M* = 0.17, SD = 0.76) and Team Gen (*M* = 0.29, SD = 1.22); *t*(573) = − 1.305, *p* = 0.01; and mean filled teeth outcomes between Team Spec (*M* = 1.03, SD = 2.10) and Team Gen (*M* = 0.44, SD = 1.44); t(573) = 3.99, *p* < 0.001. Moreover, there was a significant difference in restorative care level of Team Spec (*M* = 51.50%, SD = 43.22%) and restorative care level of Team Gen (*M* = 29.31%, SD = 39.37%); *t*(209) = 3.89, *p* < 0.001. See Table 2.

**Table 2:** dmft data and restorative care levels of two cleft teams with different strategies concerning specialized dental care between team visits

	Team Spec (*n* = 212)	Team Gen (*n* = 363)	*p*-value
dmft			
0	119 (56%)	241 (66%)	0.50
1–3	46 (22%)	51 (14%)	
> 3	47 (22%)	71 (20%)	
Mean (SD)	2.03 (3.27)	1.82 (3.58)	0.84
dt			
0	152 (72%)	265 (73%)	0.20
1–3	43 (20%)	60 (17%)	
> 3	17 (8%)	38 (10%)	
Mean (SD)	0.83 (1.78)	1.09 (2.71)	0.012*
mt			
0	197 (93%)	331 (91%)	0.19
1–3	11 (5%)	19 (5%)	
> 3	4 (2%)	13 (4%)	
Mean (SD)	0.17 (0.76)	0.29 (1.22)	0.01*
ft			
0	151 (71%)	313 (86%)	< 0.001*
1–3	31 (15%)	32 (9%)	
> 3	30 (14%)	18 (5%)	
Mean (SD)	1.03 (2.10)	0.44 (1.44)	< 0.001*
Restorative care level	51.5%	29.3%	< 0.001*

*dmft* sum of the decayed, missing, filled primary teeth, *dt* number of primary decayed teeth, *mt* number of primary missing teeth due to caries, *ft* number of primary filled teeth

*p* < 0.05 is considered significant. *Significant difference based on independent t-test for continuous outcomes and Chi square tests for categorized outcomes

Table 3 presents the results of the NBHR, examining the association between cleft team location/strategy and dental caries. There was no statistically significant difference in the prevalence of caries (zero part) between Team Spec and Team Gen in Model 1 (OR 1.17; 95% CI 0.70, 1.95), nor in Model 2 (OR 1.18; 95% CI 0.71, 1.97). However, in those patients who have caries, the team strategy was significantly associated with an increase in dmft (RR 1.50; 95% CI 1.03, 2.18). After full adjustment, the NBHR revealed a borderline significant association between Team location and dental caries experience (RR 1.45; 95% CI 1.00, 2.11).

**Table 3:** Negative binomial hurdle regression for the association between cleft team strategy and dmft

Cleft team	Model 1		Model 2	
	ZERO	COUNT	ZERO	COUNT
	OR (95% CI)	RR (95% CI)	OR (95% CI)	RR (95% CI)
Team Gen	*Reference*	*Reference*	*Reference*	*Reference*
Team Spec	0.85 (0.51–1.43)	0.67 (0.46–0.97)*	0.85 (0.51–1.41)	0.69 (0.47–1.00)

Model 1 is adjusted for age; Model 2 is adjusted for age, gender and cleft type

*dmft* sum of the decayed, missing, filled primary teeth, *OR* odds ratio, *RR* rate ratio, *CI* confidence interval

*Represents a statistical significant difference

A generalised linear model with a binomial distribution and logit link was used to analyse restorative care level in children with dmft > 0, adjusted for treatment centre, age, gender, and cleft type. Treatment at Team Spec was associated with higher odds of restoration (OR = 1.17; 95% CI 1.00, 1.38; *p* = 0.045). Age, gender, and cleft type were not statistically significantly associated with restorative care level (all *p* > 0.2), as presented in Table 4.

**Table 4:** Binomial generalized linear model for restorative care level

Variable	Category	OR (95% CI)
Cleft team	Team Gen	*Reference*
	Team Spec	1.17 (1.00–1.38)*
Gender	Female	*Reference*
	Male	0.98 (0.87–1.09)
Cleft type	CP	*Reference*
	CLA	1.05 (0.89–1.26)
	CLAP	1.09 (0.92–1.30)
Age	Continuous	0.04 (0.97–1.12)

Odds ratios (OR) derived from a generalized linear model with binomial distribution and logit link, adjusted for center, gender, cleft type and age

*Represents a significant difference

Finally, when comparing dmft data of the CL/P population (Team Spec combined with Team Gen) (*N* = 575, *M* = 1.90, SD = 3.47) with reference data from non-affected controls (*N* = 4146, *M* = 0.97, SD = 2.17)(van Meijeren-van Lunteren et al. 2021), caries experience was significantly higher in the current CL/P population, *t*(574) = 8.83, *p* < 0.001.

## Discussion

Children with cleft lip and/or palate (CL/P) treated in a cleft team that generally refers all patients for specialised paediatric dental care have a higher restorative care level and less extracted teeth due to caries compared to CL/P patients receiving general dental care. However, even with specialised paediatric dental care, caries prevention remains challenging as the prevalence of dental caries among CL/P patients remains elevated compared to their unaffected peers.

In the current study, we reported dmft levels among Dutch CL/P patients that were lower than those reported in the Cleft Care UK Study. Smallridge et al. (2015) reported a mean dmft of 2.3 amongst 5-year-old CL/P patients in the UK, with 48% of children being caries-free. However, the UK study did not specify whether the children received care from general or specialised dental care professionals.

Maintaining good oral health is important for CL/P patients, but unfortunately caries prevention appears to be challenging in this group. CL/P patients are more likely to experience dental anxiety and to require general anaesthesia for dental treatment, leading to additional health risks and financial costs. For example, Lehtonen et al. (2015) reported that 17.5% of CL/P patients required general anaesthesia for dental procedures, compared to 0.2% among non-CL/P children. The Team Spec strategy was not associated with lower sum dmft scores, meaning that caries prevention remains challenging for CL/P patients even with specialist advice and care. However, besides higher filled teeth scores, Team Spec reported lower missing teeth scores too; which might suggest that restorative interventions are being implemented in a timely manner at Team Spec, prior to the progression of carious lesions to a severity that tooth extraction is inevitable. Comparably, the NBHR in the current study found higher estimates in the mean number of teeth affected by dental caries in the Team Gen compared to Team Spec, and although the association was no longer statistically significant after accounting for covariates, a clinical effect should not be ruled out. Moreover, the present results indicated that, after controlling for relevant demographic and clinical factors, the cleft centre was the primary factor associated with differences in restorative care level.

However, the findings of the present study must be considered in the context of their limitations. There was a notable age difference between the study groups, which is relevant because caries experience is cumulative, and scores tend to increase with age during early childhood and could decline when primary teeth exfoliate (Worth et al. 2017). In the present sample, most dmft assessments in the Team Spec group were conducted around age 6 years, whereas those in the Team Gen group were collected around age 4 years. Statistical correction for age was applied, and the differences in caries experience and restorative care level remained. Notably, CL/P patients are known for their delay in tooth development; therefore, most CL/P patients aged 6 years are not yet in the first transition phase (Van Dyck et al. 2019).

Whilst information on the protocols regarding dental care strategy of the included cleft teams was known, there was no detailed information available on the exact number of patients receiving specialised versus general dental care within each team. Some children may have received specialised care, despite being classified under the general care category and vice versa. This constitutes misclassification and represents a form of information bias, which may have led to an underestimation of the true strength of existing associations. Moreover, several known caries risk factors in CL/P patients, such as parental education level and parental ethnicity, were not included in the present study, potentially increasing the risk of confounding (van der Knaap-Kind et al. 2026). But also, information on nutritional guidance, feeding strategies, surgical history, quality of the enamel and caregiver-led oral hygiene practices was not included.

Additionally, dmft is a quick and simple tool, valuable for broad surveillance, but limited in clinical nuance. Being based on visual examination, dmft often underreports hidden or proximal carious lesions (Gudipaneni et al. 2022), although Cohen’s Kappa showed good scores for reliability, but does not account for non-restorative caries treatment (NRCT), which is a common approach in modern caries management in the Netherlands. The aim of NRCT is to inactivate the caries process by disturbing the bacterial biofilm to prevent acid production from reaching a level resulting in net loss of mineral from the tooth. The non-restorative approach is a treatment strategy that may be less demanding for a child, but is reliant on behaviour changes to reduce caries risk. Delay or replacement of invasive restorative treatment by a preventive approach is said to increase comfort for children and promotes oral health over time (Ekstrand et al. 2009; Jasulaityte and Burgersdijk 2021). When using the dmft index for quantifying caries experience, it is crucial to make agreements on how to score teeth that are successfully and unsuccessfully treated with NRCT. Uniformity in scoring protocols across cleft centres is essential to ensure consistency and comparability in outcomes. Future meetings between dentists of the cleft teams internationally are required to reach a consensus. Besides this, narrowing the age range for the dmft index could be considered as a recommendation for the ICHOM cleft set, to improve comparability and precision of caries outcome measurements.

Finally, further research is warranted to gain a deeper understanding of the cost-effectiveness of the two treatment strategies, thereby enabling evidence-based decision-making in clinical practice. In the future, larger sample sizes may allow for further differentiation between unilateral and bilateral clefts and it may also be valuable to include a site-specific caries lesion analysis.

Considering the limitations of the present study, it is concluded that having not only a paediatric dentist present at the cleft team, but also available for dental care between team visits, leads to a higher restorative dental care level and less extracted teeth due to caries, in patients born with a cleft lip and/or palate (CL/P). Therefore, CL/P patients receiving specialised dental care will also experience fewer negative consequences of severe untreated dental caries, such as pain and infections (Narbutaite et al. 2024). For the future, further research is necessary on cost analysis of standard treatment by a paediatric dentist for CL/P patients, and on improving preventive dental care for this vulnerable group of children.

## Why this paper is important to paediatric dentists

- More than 20% of CL/P patients experience severe caries (dmft > 3).
- Receiving dental care from a specialised dentist between cleft team visits is beneficial for the restorative care level of CL/P patients.
- Effective preventive dental care remains difficult to provide to CL/P patients.

## Data Availability

Not all data are publicly available, but can be accessed upon request.
